# Prevalence of Metabolic Syndrome among Apparently Healthy Adult Population in Pakistan: A Systematic Review and Meta-Analysis

**DOI:** 10.3390/healthcare11040531

**Published:** 2023-02-10

**Authors:** Syed Omair Adil, Md Asiful Islam, Kamarul Imran Musa, Kashif Shafique

**Affiliations:** 1 Department of Community Medicine, School of Medical Sciences, Universiti Sains Malaysia, Kubang Kerian 16150, Kelantan, Malaysia; 2 School of Public Health, Dow University of Health Sciences, Karachi 75330, Pakistan; k.shafique@duhs.edu.pk; 3 WHO Collaborating Centre for Global Women’s Health, Institute of Metabolism and Systems Research, University of Birmingham, Birmingham B15 2TT, UK; m.a.islam@bham.ac.uk

**Keywords:** metabolic syndrome, Pakistan, prevalence

## Abstract

Background: Metabolic syndrome (MetS) is considerably higher worldwide. It varies greatly in different populations geographically and based on criteria used to diagnose the disease. This review was conducted to determine the prevalence of MetS among apparently healthy adults of Pakistan. A systematic review was performed on Medline/PubMed, SCOPUS, ScienceDirect, Google Scholar, and Web of Science databases until July 2022. Articles published on Pakistani healthy adult population reporting MetS were included. Pooled prevalence was reported with 95% confidence interval (CI). Of 440 articles, 20 articles met the eligibility. Results: The pooled prevalence of MetS was 28.8% (95% CI: 17.8–39.7). The maximum prevalence was from a sub-urban village of Punjab (68%, 95% CI: 66.6–69.3) and Sindh province (63.7%, 95% CI: 61.1–66.3). International Diabetes Federation guidelines had shown 33.2% (95% CI: 18.5–48.0) whereas National Cholesterol Education Program guidelines showed 23.9% (95% CI: 8.0–39.8) prevalence of MetS. Furthermore, individuals with low high-density lipoprotein (HDL) 48.2% (95% CI: 30.8–65.6), central obesity 37.1% (95% CI: 23.7–50.5), and high triglyceride 35.8% (95% CI: 24.3–47.3) showed higher prevalence. Conclusion: A considerably higher prevalence of MetS was observed among apparently healthy individuals in Pakistan. High triglyceride, low HDL, and central obesity were found as significant risk factors. (Registration # CRD42022335528)

## 1. Introduction

Non-communicable diseases (NCDs) are a great threat to public health [1]. Various studies have been conducted that reported the burden of Metabolic Syndrome (MetS), which is a cluster of non-communicable conditions including insulin resistance, hypertension, and hyperlipidemia, in disease-specific adult populations and also in general population [2,3,4,5,6]. As per the estimates, one quarter of the population in the Unites States and Europe are affected by MetS, while the pooled prevalence in South Asian population ranged from 14% to 32.5% [7]. Although, the burden of the MetS is considerably higher worldwide. It varies greatly in different populations geographically and on the basis of criteria used to diagnosed MetS [8,9]. Currently, different definitions are being used for the diagnosis of MetS, which include National Cholesterol Education Program for Adult Treatment Panel (NCEP-ATP) III, International Diabetes Federation (IDF), modified NCEP-ATP III, and Harmonized criteria by Joint Interim Societies [5]. Though, the main components used for the diagnosis of MetS in each definition is similar, the prevalence of MetS varies widely depending upon the criteria used for diagnosis.

As discussed, the magnitude of the problem varies greatly in terms of geographic representation of the individuals too. The prevalence of MetS and its associated risk factors are highly prevalent in Pakistan as well. A recently published study on 15,590 high-risk citizens of Pakistan has reported MetS prevalence as 54.9% using IDF and 55.4% using NCEP-ATP III criteria [10]. Another study carried among 4319 individuals have reported 68.13% prevalence [11].

A number of studies have been published internationally that have reported country-specific pooled prevalence of MetS [12,13,14,15]. However, a thorough literature search has revealed that no attempt has been made to estimate the pooled prevalence of MetS from Pakistan. Keeping in view the increasing burden of the risk of MetS and its components in Pakistan, there is a dire need of systematically reviewing the evidence to determine the overall prevalence of MetS based on published studies among the Pakistani population. In addition, the lack of adequate surveillance system or registry to monitor the magnitude of the problem has also created the need of such a type of study from this part of world. Therefore, we conducted a systematic review and meta-analysis of published studies that have reported the prevalence of MetS in then Pakistani adult general population who perceived themselves as apparently healthy based on the absence of any disease. The outcome of the study will equally be beneficial for the policymakers and healthcare providers in making the national policies and guidelines to prevent the increasing burden and consequence of MetS in Pakistan.

## 2. Materials and Methods

This systematic review was performed based on the PRISMA (Preferred Reporting Items for Systematic Reviews and Meta-Analyses) Statement. Moreover, the proposal was registered on the International Prospective Register of Systematic Reviews (PROSPERO) database prior to conducting the review (Registration # CRD42022335528).

### 2.1. Search Strategy

Systematic search was performed on Medline/PubMed, SCOPUS, ScienceDirect, Google Scholar, and Web of Science databases for the articles published until July 2022 without time filtering. In addition to the mentioned databases, reference lists of recent and relevant systematic reviews/meta-analysis and references cited in the relevant original articles were also screened for studies not found in the main search strategy. The search term used are mentioned in detailed in Supplementary Table S1. The final search was conducted by combining individual search results by using appropriate Boolean operators (“OR” and “AND”). The search results were imported into EndNote^®^ version X7 software (Thomson Reuters, New York, NY, USA) in .enl format and any duplicate studies was removed.

### 2.2. Eligibility Criteria

The systematic review included cross-sectional, case-control or cohort study design articles that have reported the prevalence of MetS in healthy Pakistani adults as an endpoint.

Pakistani adults aged 18 years or above of any gender without any disease or disorder were included. The systematic search did not restrict articles based on geographic region such as rural or urban or study setting such as community facility or workplace. However, articles with disease-specific populations such as schizophrenia, polycystic ovarian syndrome, or rheumatoid arthritis, articles with no accessibility to the full text, language other than English, publication, and articles other than original research, i.e., symposium or conference abstracts, book chapters, review papers, case reports or letters to the editor were excluded. Furthermore, only those studies were included in which MetS was diagnosed using any one of the four internationally recognized criteria, i.e., National Cholesterol Education Program Adult Treatment Panel (ATP) III guidelines, International Diabetes Federation guidelines, Modified ATP III criteria, or Harmonized Asia Pacific criteria. The details of the four criteria are mentioned in supplementary Table S2.

### 2.3. Selection of Studies

The literature search was carried out independently by SOA and MAI, who also reviewed the title, abstract, and keywords of every study found for potential inclusion in the review. For the studies that were deemed to be pertinent, the full text of the article was retrieved. Two researchers (SOA and MAI) separately conducted additional screening of the abstracts and full texts of the retrieved articles to identify the studies that met the requirements for the current review. Any differences between two authors during the entire selection process were settled by consensus or by consulting a third investigator (KIM). The third investigator supervised the overall review process’s quality (KIM).

### 2.4. Data Extraction and Management

From the included studies, two investigators (SOA and MAI) retrieved the pertinent study features for the review and entered them into a predetermined Excel spreadsheet. In order to assure consistency, rule out bias, and reduce errors, SOA and MAI double-checked the extracted data before it was included in the review. The following information was taken:

1. General Information: Author, Study title, Publication year.
2. Methods section: Study design, province, community (urban/sub-urban/rural), study period, study setting, population, sample size, age group, sampling technique, and diagnostic criteria.
3. Outcome section: Prevalence of MetS.

### 2.5. Risk of Bias Assessment in Included Studies

The risk of bias/quality assessment was performed using Joanna Briggs Institute (JBI) appropriate critical appraisal tools by two investigators (SOA and MAI) for cross-sectional and case-control studies. The checklist for cross-sectional studies consisted of eight items with three options: ‘yes’, ‘no’, and ‘unclear’. While the checklist for case-control studies consisted of ten items with three options: ‘yes’, ‘no’, and ‘unclear’. The percentage of “yes” responses was used to determine each article’s final score. When the overall score was 49% or below, between 50% and 69%, 70% or above, studies were categorized as having a high, moderate, or low risk of bias, respectively. The high-risk was labeled as a poor-quality paper, moderate as moderate quality, and low-risk as a high quality of paper.

### 2.6. Statistical Analysis

The pooled prevalence and 95% CIs of MetS among apparently healthy individuals living in Pakistan were calculated. A random-effect model was used for the calculations in all analyses. The I2 statistic was used to determine study heterogeneity (I2 > 75% showed considerable heterogeneity) and the significance level was presented according to Cochran’s Q test. Subgroup analysis was also performed as (A) diagnostic criteria for MetS; and (B) individual components of MetS. In order to test the results’ robustness, sensitivity analyses were carried out excluding low or moderate quality studies and excluding outlier studies. Prevalence estimates were plotted against standard errors in a funnel plot to measure publication bias, and Egger’s test was used to validate funnel plot asymmetry. Outlier studies and potential sources of heterogeneity were detected by constructing a Galbraith plot. Metaprop function in meta package (version 4.15-1) were used to generate the analyses and plots and the metafor (version 2.4-0) package of R (version 3.6.3) in RStudio (version 1.3.1093) were utilized.

## 3. Results

**Figure 1:**
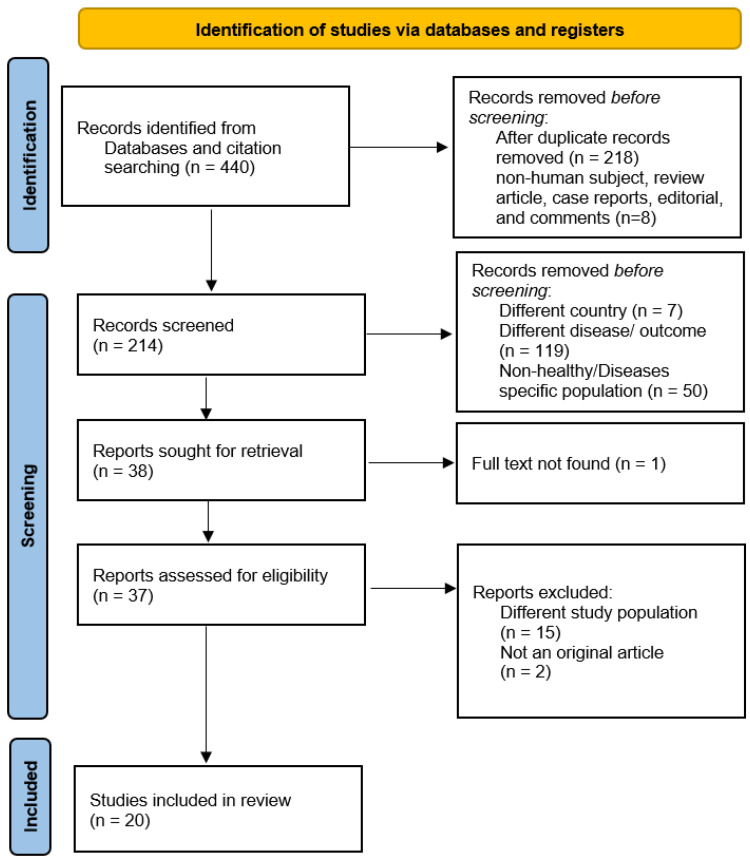
Flow Chart showing the search strategy and selection of studies. Adapted from Ref. [33]. For more information, visit: http://www.prisma-statement.org/ (accessed on 6 February 2023).

Initially, 440 articles were found from five databases. Of these, 226 articles were removed because of duplication and articles other than original research paper. Another 119 studies were excluded from the remaining articles based on different disease/outcome. Furthermore, seven articles were excluded with reasons of being populations from different country. Lastly, one article was removed due to the non-availability of full text. Finally, the systematic review and meta-analysis comprised 20 publications that matched the eligibility requirements [9,10,11,16,17,18,19,20,21,22,23,24,25,26,27,28,29,30,31,32]. (Figure 1).

### 3.1. Characteristics of Included Studies

This meta-analysis is based on a study of 30,419 apparently healthy individuals. Most of the studies, 17 (85.0%), were cross-sectional in nature. The majority of the studies were carried out in Sindh Province and Punjab province, i.e., 11 (55.0%) and 6 (30.0%), respectively, while in most the areas were urban, 17 (85.0%). There were two studies that were conducted only on female populations, of which one included apparently healthy pregnant women. The minimum samples included in the study was 60, whereas maximum sample size was 15,590. Most of the studies, eight (40.0%), have included only IDF criteria for diagnosis of MetS followed by NCEP-ATP III 7 (35.0%). In four (20.0%) studies, MetS was screened using multiple diagnostic criteria, while in one study diagnostic criteria was not reported. The detailed characteristics of the included studies are summarized Table 1.

### 3.2. Primary Outcomes

**Figure 2:**
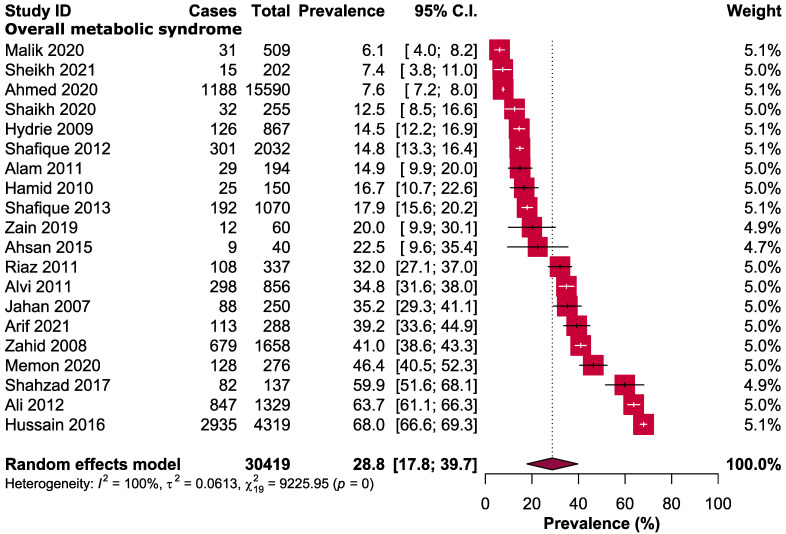
Prevalence of metabolic syndrome in Pakistan.

The overall pooled prevalence of MetS among the apparently healthy adult population of Pakistan was 28.8% (95% CI: 17.8–39.7). (Figure 2) Province-wise analysis showed that a sub-urban village of Punjab reported the highest prevalence of MetS (68%, 95% CI: 66.6–69.3), followed by Sindh (63.7%, 95% CI: 61.1–66.3), Baluchistan (59.9%, 95% CI: 51.6–68.1) whereas the lowest prevalence was observed from two studies of Punjab in which the reported prevalence was 6.1% (95% CI: 4.0–8.2) and 7.4% (95% CI: 3.8–11), respectively.

### 3.3. Subgroup Analysis

Stratification on the basis of diagnostic accuracy has shown that a total of 12 studies have utilized IDF criteria for diagnostic of MetS in Pakistan either as single or in combination of other methods. The findings of the IDF have shown 33.2% (95% CI: 18.5–48.0) prevalence of MetS in apparently healthy individuals of Pakistan. Whereas 10 studies have utilized NCEP guidelines for the diagnosis of MetS either as single or in combination of other methods. The findings of the NCEP have shown prevalence of MetS in 23.9% (95% CI: 8.0–39.8) of apparently healthy individuals of Pakistan.

Further subgroup analysis was performed to see component-wise distribution of MetS. The findings revealed that among individuals who had central obesity, the prevalence for MetS was found to be 37.1% (95% CI: 23.7–50.5). In hypertensive patients, the MetS prevalence was found to be 29.5% (95% CI: 20.0–38.9). In individuals with increased glucose level, the MetS prevalence was found to be 20.6% (95% CI: 15.3–25.9). In individuals with high triglyceride, the MetS prevalence was found to be 35.8% (95% CI: 24.3–47.3). In individuals with low HDL level, the MetS prevalence was found to be 48.2% (95% CI: 30.8–65.6). Table 2, Figure S1.

### 3.4. Quality Assessment and Publication Bias

**Figure 3:**
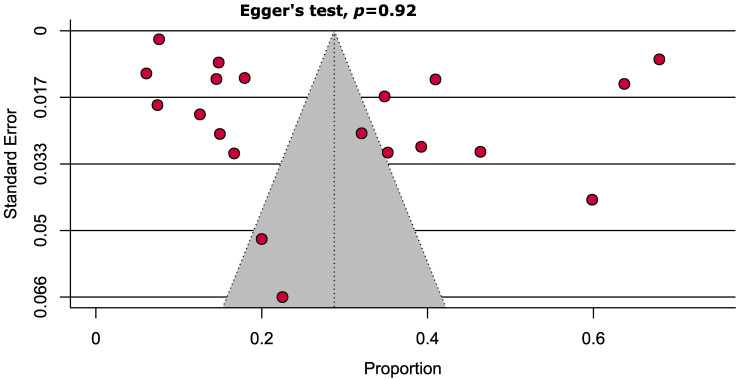
Funnel plot representing the prevalence of metabolic syndrome in Pakistan shows absence of significant publication bias.

The assessment of publication bias was carried out using Egger’s test. There were no insignificant study effects, and the absence of publication bias is demonstrated by the non-significant *p*-value (*p* = 0.920). Figure 3 uses a funnel plot to graphically describe the test for publication bias. The JBI findings for assessment of risk of bias/quality assessment showed six papers with low/moderate quality whereas majority, i.e., 14, were high-quality papers. (Tables S3 and S4)

### 3.5. Sensitivity Analyses

**Figure 4:**
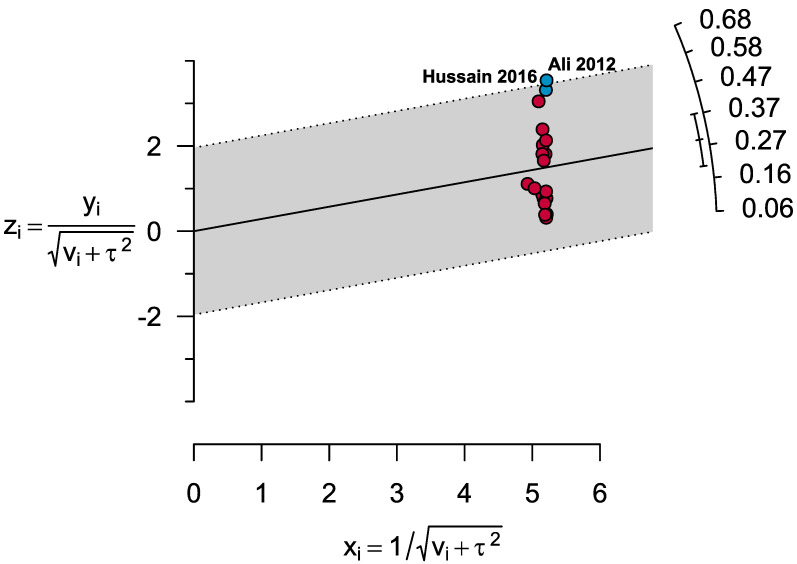
Galbraith plot shows two outlier studies.

The Galbraith plot identified Ali 2012 and Hussain 2016 as the outlier studies that reported MetS in apparently healthy individuals from Punjab province (Figure 4).

After excluding these outlier studies, the prevalence of MetS was found to be 24.4% (95% CI: 18.6–30.2). This prevalence was 4.4% higher compared to the pooled prevalence of MetS. The maximum prevalence after excluding outlier studies was observed in a study conducted in Baluchistan province 59.9% (95% CI: 51.6–68.1), while the lowest prevalence was observed in a study from Punjab province 6.1% (95% CI: 4.0–8.2). After excluding the two studies with small sample size (n < 100), the MetS prevalence was found to be 29.6% (95% CI: 18.0–41.1). This prevalence was 0.8% higher compared to the pooled prevalence of MetS. The maximum prevalence after excluding studies with small sample was observed in a study carried out in a sub-urban village of Baluchistan province 68.0% (95% CI: 66.6–69.3) while the lowest prevalence was observed in a study from Punjab province 6.1% (95% CI: 4.0–8.2). After excluding the six low and moderate quality studies, the MetS prevalence was found to be 27.0% (95% CI: 18.0–36.0). This prevalence was 1.8% lower compared to the pooled prevalence of MetS. The maximum prevalence after excluding studies with low and moderate qualities was observed in a study conducted in an urban city of Sindh province 63.7% (95% CI: 61.1–66.3), while the lowest prevalence was observed in a study from Punjab province 6.1% (95% CI: 4.0–8.2). Table 3, Figure S2.

## 4. Discussion

We have performed this review and meta-analysis to report the prevalence of MetS in the Pakistani population. The country-wise estimates for MetS in healthy populations are available from other countries but such studies are not reported from Pakistan that has performed a thorough review on studies reporting the MetS prevalence in apparently healthy Pakistani adult population. In the current study, the overall burden of MetS along with the province-wise estimates are reported. In addition to this, we have also reported prevalence of MetS based on the presence of each component of MetS. In total, we analyzed data from 20 studies with 30,419 participants. The province of Sindh conducted the majority of the studies, followed by the province of Punjab. Most of these studies were carried out in an urban setting with consecutive sampling technique. There were five studies that have been conducted on more than thousands of individuals. IDF and NCEP are the diagnostic criteria of MetS that were predominantly used in studies. However, a study has reported burden of apparently healthy individuals using AHA and WHO criteria along with IDF and NCEP. Low risk of bias existed in the majority of the examined studies.

The findings of the current systematic review and meta-analysis revealed that the prevalence of MetS among apparently healthy adult Pakistani individuals was 28.8%. This prevalence is somewhat similar with the previously published reviews on MetS from other countries [13,34,35,36]. In particular, a recently published review from neighboring country of Pakistan, i.e., India which shares much similar biological, physical, and environmental characteristics, has reported pooled prevalence quite similar to Pakistan, i.e., 30% [13]. The pooled prevalence of MetS in the current study among the healthy adult population of Pakistan is slightly higher from a previously published review from China in which the pooled prevalence of MetS in the healthy adult population was 22% [34]. However, the author stated that this prevalence in China was more than half almost two decades ago, i.e., year-wise stratification has revealed 8.8% prevalence of MetS in 1990s that increases to 29.3%, as reported by the findings of Chinese studies carried out in between 2011 to 2015 [34]. Similar findings were also reported from another neighboring country Iran in which MetS was 23.8% in 2018, which later increased to 30.4% [35,36]. Similar findings were also reported from another Asian country, i.e., Bangladesh in which the pooled prevalence was also 30%, However, in Vietnam, the pooled prevalence was reported quite low, i.e., 16.1% [37,38]. While the findings of studies that have reported pooled prevalence from other regions showed pooled prevalence of 24.9% in Latin America, 54% in Mexico, and 21.2% in Ghana [39,40,41].

High prevalence of MetS was reported from sub-urban village of Punjab Province and urban city Karachi, Pakistan, which is in Sindh province. Both studies have ample number of samples (more than thousands of samples) and MetS prevalence was estimated using IDF diagnostic criteria [11,32]. The high prevalence in a sub-urban village of Punjab province is an alarming issue that highlighting the need of implementation of strict measures to combat the prevalence of disease. Whereas Karachi being a metropolitan city of Pakistan with so much cultural, social, and wide geographical boundaries needs further studies to more closely evaluate the individuals who are more prone to the development of MetS. In addition to this, the uncontrolled and unplanned increase in the population of Karachi city has made it difficult to identify the at-risk individuals and identification of potential risk factors that are causing the increasing prevalence of the disease. However, common factors such as low physical activity, psychosocial stress, and unhealthy diet and lifestyle are some of the factors that may be responsible for the increasing prevalence of the disease in the apparently healthy adult population of Karachi city.

Unfortunately, there is no previous study from Pakistan that has estimated the pooled prevalence of MetS; therefore, we are unable to predict this prevalence is increased or decreased. However, there is a wide variation observed in the reported prevalence of MetS in studies from Pakistan. The excessive variation in the prevalence could be due to the urbanization and industrialization in the country, which eventually effect lifestyle and dietary habits [42,43,44]. One more important point that needs consideration is the finding of the subgroup analyses estimating the pooled seroprevalence of metabolic syndrome in Pakistan. The pooled seroprevalence was higher in individuals who had central obesity and dyslipidemia. The reason for the higher prevalence may be same as discussed earlier, i.e., low physical activity, unhealthy diet, and lifestyle. All these factors are known contributing factors to central obesity and dyslipidemia, which results in increasing prevalence of MetS too. Moreover, after the occurrence of the COVID-19 outbreak, the increasing risk of these sedentary and unhealthy activities among apparently healthy individuals has increased. A decline in physical activity during the movement restriction period in the COVID period was widely noticed. It is important to point out that as visceral and centripetal obesity induces a low-grade pro-inflammatory process with an excessive inflammatory cytokine and adipokines, it leads to insulin resistance, high blood pressure and dyslipidemia; thus, the severity increases with increased weight. There is a need of further community-based screening studies for metabolic syndrome and associated risk factors among apparently healthy individuals to identify the burden of the disease after the COVID outbreak.

The main strength of the study is that it presents the first in-depth analysis of the prevalence of MetS in healthy adult Pakistanis that appear to be in good health. This systematic review and meta-analysis have only included studies in which screening for MetS was performed in individuals who were unaware of any component of the MetS, and they were perceiving themselves as healthy. Moreover, estimates based on the presence of specific MetS indicators and diagnostic criteria were also reported in the study. The current review did not exhibit any discernible publication bias. However, there are certain limitations in our review. Summarizing and reporting the burden of MetS in more than one quarter of the apparently healthy adult individuals in Pakistan is difficult because of the usage of different diagnostic criteria by most of the studies. By doing subgroup analysis based on diagnostic standards and the existence of MetS characteristics, we have attempted to overcome this limitation. However, the pooled estimates of the sub-group analysis have revealed major variation in the prevalence of MetS.

Despite of these limitations, this study has reported important baseline information on the burden of MetS among apparently healthy adult population of Pakistan. The current study has reported high prevalence among apparently healthy individuals who had low HDL, high triglyceride, and central obesity. The results of the current review show that Pakistan has a serious public health issue with MetS. The government must set aside sufficient funds and implement appropriate initiatives to address the burden of MetS in the country. In this regard, population-level screening for these non-communicable diseases is of utmost importance. To better manage MetS, a well-rounded, comprehensive healthcare strategy involving several levels of healthcare is required. These approaches would allow us to lessen the fatality caused by each of the individual components of MetS as well as the financial burden associated with these disorders. To determine changes in the prevalence and determinants causing the incidence of MetS, meta-analyses of population-based studies are required in future.

## 5. Conclusions

MetS is considerably prevalent among the Pakistani population who perceived themselves as healthy based on the absence or ignorance with the silent diseases such as obesity, dyslipidemia and hypertension. In particular, prevalence was significantly higher among individuals who are living in Sindh and Punjab province. Moreover, individuals having high triglyceride, low HDL, and central obesity are at significant risk for development of MetS.

## Figures and Tables

**Table 1:** Major Characteristics of the Included Studies.

Study ID [References]	Study Design	Province	Community	City/Village	Study Period	Study Setting	Sample Size	Age Group	Sampling Technique	Diagnostic Criteria
Ahmed 2020 [10]	Cross-sectional	Sindh	Urban	Karachi	Not reported	Community	15,590	30–64 years	Non-probability consecutive	IDF NCEP ATP III
Ahsan 2015 [24]	Cross-sectional	Sindh	Urban	Karachi	Jan–Aug 2008	Facility	40	30 years and above	Systematic random technique	NCEP ATP III
Alam 2011 [20]	Cross-sectional	Punjab	Urban	Bhawalpur	June 2008 to May 2009	Facility	194	20–60 years	Simple Random Sampling	NCEP ATP III
Ali 2012 [32]	Cross-sectional	Sindh	Urban	Karachi	July to Dec 2011	Facility	1329	18 years and above	Non-probability convenience sampling	IDF
Alvi 2011 [26]	Cross-sectional	Sindh	Urban	Karachi	July to Dec 2004	Community	867	25 years and above	Simple Random Sampling	IDF
Arif 2021 [28]	Cross-sectional	KPK	Urban	Peshawar	Not reported	Community	288	20 years and above	Multistage Clustering Sample	IDF
Hamid 2010 [21]	Case-Control	KPK	Urban	Peshawar	2006	Community	150	40 years and above	Non-probability convenience sampling	NCEP ATP III
Hussain 2016 [11]	Cross-sectional	Punjab	Sub-Urban Village	Kacha, Dera Chahal, Shadawal and Samsani Khui	Not Reported	Community	4319	Adults (Exact age not mentioned)	Convenience sampling	IDF NCEP ATP III AHA WHO
Hydrie 2009 [9]	Cross-sectional	Sindh	Urban	Karachi	July 2004, to Dec 2004	Community	363	25 years and above	Random Sampling	IDF Modified NCEP ATP III
Jahan 2007 [27]	Cross-sectional	Sindh	Urban	Karachi	Dec 2004 to Apr 2005	Facility	250	25 years and above	Convenience sampling	NCEP ATP III
Malik 2020 [16]	Cross-sectional	Punjab	Urban	Lahore	June 2016 to March 2017	Facility	509	18–24 years	Convenience sampling	IDF
Memon 2020 [30]	Cross-sectional	Sindh	Urban	Hyderabad	Jan 2018 to June 2018	Facility	276	35–60 years	Convenience sampling	NCEP ATP III
Riaz 2011 [25]	Cross-sectional	Sindh	Urban	Karachi	Not Reported	Community	337	25 years and above	Random Sampling	IDF
Shafique 2012 [19]	Cross-sectional	Punjab	Urban/Rural Both	Faisalabad /Peripheral area	Jan 2006 to June 2009	Community	2032	30–75 years	Convenience sampling	IDF
Shafique 2013 [22]	Cross-sectional	Sindh	Urban	Karachi	Not Reported	Community	1070	16–75 years	Simple Random Sampling	IDF
Shaikh 2020 [18]	Cross-sectional	Sindh	Urban	Hyderabad	March to Sep 2019	Facility	137	25–65 years	Purposive sampling	NCEP ATP III
Shahzad 2017 [31]	Cross-sectional	Baluchistan	Urban	Quetta	Aug 2015 to Jan 2016	Facility	255	17–19 years	Convenience sampling	IDF
Sheikh 2021 [17]	Case-Control Study	Punjab	Urban	Lahore	Oct 2017 to March 2018	Facility	202	20–36 years	Convenience sampling	NCEP ATP III
Zahid 2008 [29]	Cross-sectional	Punjab	Rural	Kharian	March to July 2006	Community	160	20 years and above	Random sampling	IDF Modified NCEP ATP III
Zain 2019 [23]	Case-Control	Sindh	Urban	Hyderabad	March 2015 to Sep 2015	Facility	60	19–40 years	Consecutive sampling	Not Reported

**Table 2:** Subgroup analyses estimating the pooled seroprevalence of metabolic syndrome in Pakistan.

Subgroups	Pooled Prevalence [95% CIs] (%)	Number of Studies Analyzed	Total Number of Patients
**Diagnostic criteria**			
IDF	33.2 [18.5–48.0]	12	28,992
NCEP	23.9 [8.0–39.8]	10	22,143
**Metabolic syndrome types**			
Central obesity	37.1 [23.7–50.5]	15	25,331
High blood pressure	29.5 [20.0–38.9]	15	25,608
Increased glucose	20.6 [15.3–25.9]	16	26,989
High TG	35.8 [24.3–47.3]	16	26,989
Low HDL	48.2 [30.8–65.6]	17	27,326

CIs: confidence intervals, IDF: International Diabetes Federation, NCEP: National Cholesterol Education Program, TG: Triglyceride.

**Table 3:** Sensitivity analyses.

Strategies of Sensitivity Analyses	Prevalence [95% CI]	Difference of Pooled Prevalence Compared to the Main Result	Number of Studies Analyzed	Total Number of Participants	Heterogeneity	
					*I^2^*	*p*-Value
Excluding outlier studies	24.4 [18.6–30.2]	4.4% lower	18	24,771	99%	<0.0001
Excluding small studies (n < 100)	29.6 [18.0–41.1]	0.8% higher	18	30,319	100%	<0.0001
Excluding low- and moderate-quality studies	27.0 [18.0–36.0]	1.8% lower	14	25,062	100%	<0.0001

CI: Confidence interval.

## Data Availability

Not applicable.

## Supplementary Materials

The following supporting information can be downloaded at: https://www.mdpi.com/article/10.3390/healthcare11040531/s1, Figure S1: Subgroup analyses based on the diagnostic criteria (A–B) and types of metabolic syndrome (C–G). Figure S2: Sensitivity analyses. Table S1: Search strategy used for PubMed. Table S2: Criteria for diagnosis of metabolic syndrome. Table S3: Quality assessment of the included case-control studies. Table S4: Quality assessment of the included cross-sectional studies.
