# Supplementary material for: Prevalence of Metabolic Syndrome among Apparently Healthy Adult Population in Pakistan: A Systematic Review and Meta-Analysis
Source: Healthcare (Basel). 2023 Feb 10;11(4):531. doi: 10.3390/healthcare11040531 (PMC9957355; doi:10.3390/healthcare11040531)
Supplement: Supplementary file 1 [file healthcare-11-00531-s001.zip › Figure S1_Subgroup analyses.pdf]

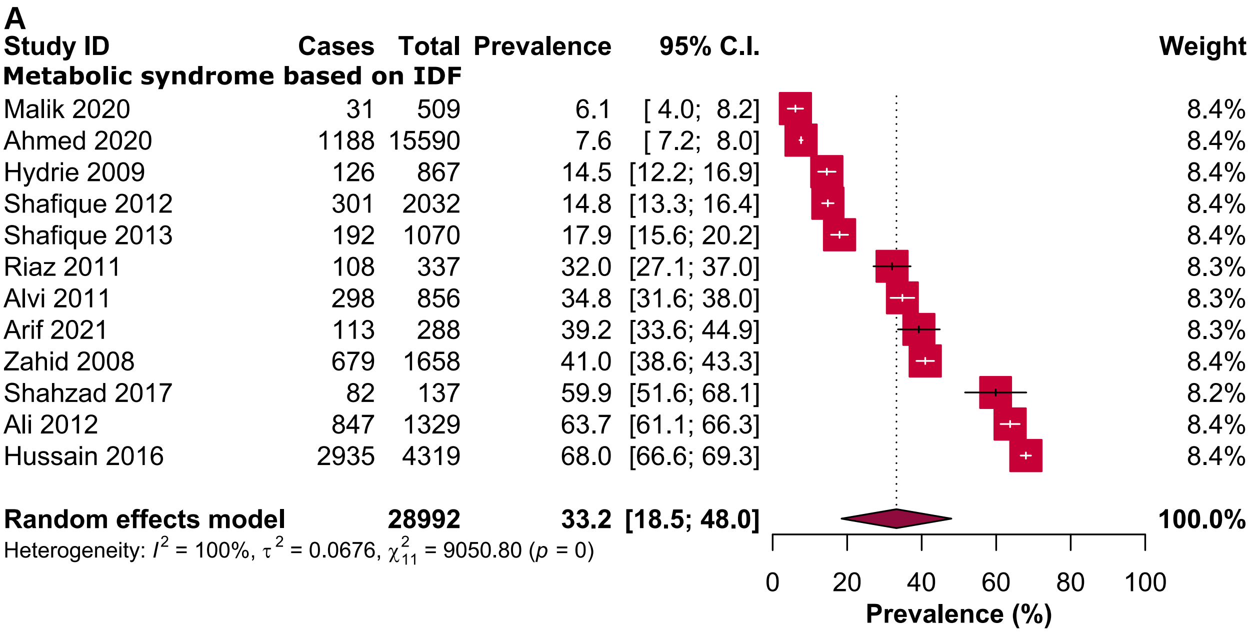
A
Study ID Cases Total Prevalence 95% C.I. Weight
Metabolic syndrome based on IDF
Malik 2020 31 509 6.1 [4.0; 8.2] 8.4%
Ahmed 2020 1188 15590 7.6 [7.2; 8.0] 8.4%
Hydrie 2009 126 867 14.5 [12.2; 16.9] 8.4%
Shafique 2012 301 2032 14.8 [13.3; 16.4] 8.4%
Shafique 2013 192 1070 17.9 [15.6; 20.2] 8.4%
Riaz 2011 108 337 32.0 [27.1; 37.0] 8.3%
Alvi 2011 298 856 34.8 [31.6; 38.0] 8.3%
Arif 2021 113 288 39.2 [33.6; 44.9] 8.3%
Zahid 2008 679 1658 41.0 [38.6; 43.3] 8.4%
Shahzad 2017 82 137 59.9 [51.6; 68.1] 8.2%
Ali 2012 847 1329 63.7 [61.1; 66.3] 8.4%
Hussain 2016 2935 4319 68.0 [66.6; 69.3] 8.4%
Random effects model 28992 33.2 [18.5; 48.0] 100.0%
Heterogeneity: I^2 = 100%, τ^2 = 0.0676, χ^2_{11} = 9050.80 (p = 0)
0 20 40 60 80 100
Prevalence (%)

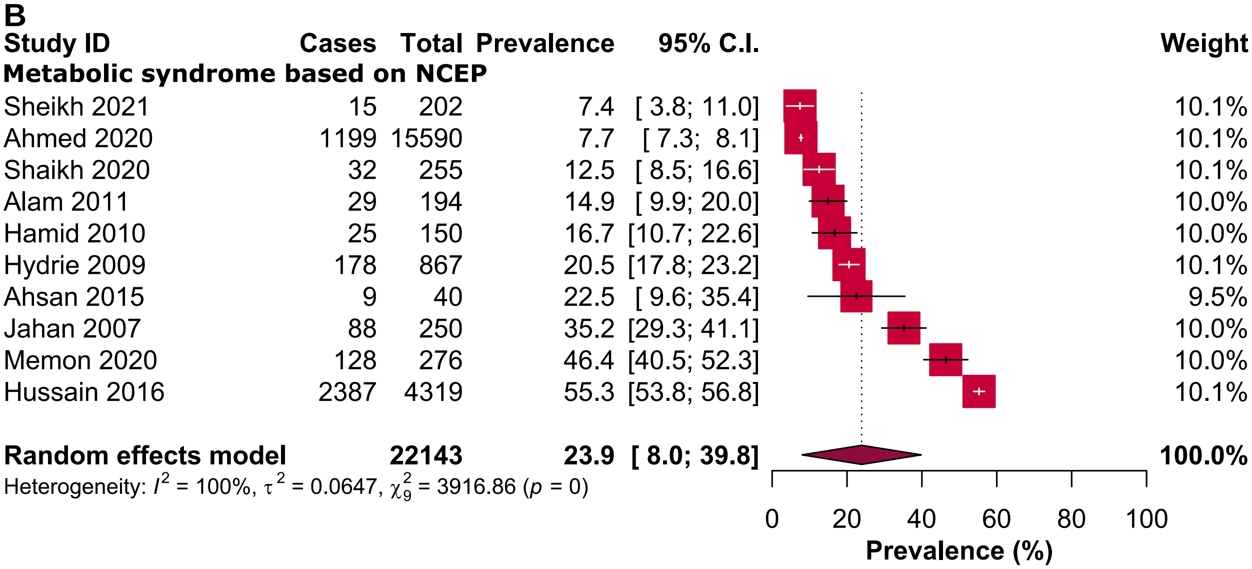
B
Study ID Cases Total Prevalence 95% C.I. Weight
Metabolic syndrome based on NCEP
Sheikh 2021 15 202 7.4 [3.8; 11.0] 10.1%
Ahmed 2020 1199 15590 7.7 [7.3; 8.1] 10.1%
Shaikh 2020 32 255 12.5 [8.5; 16.6] 10.1%
Alam 2011 29 194 14.9 [9.9; 20.0] 10.0%
Hamid 2010 25 150 16.7 [10.7; 22.6] 10.0%
Hydrie 2009 178 867 20.5 [17.8; 23.2] 10.1%
Ahsan 2015 9 40 22.5 [9.6; 35.4] 9.5%
Jahan 2007 88 250 35.2 [29.3; 41.1] 10.0%
Memon 2020 128 276 46.4 [40.5; 52.3] 10.0%
Hussain 2016 2387 4319 55.3 [53.8; 56.8] 10.1%
Random effects model 22143 23.9 [8.0; 39.8] 100.0%
Heterogeneity: I^2 = 100%, τ^2 = 0.0647, χ^2_9 = 3916.86 (p = 0)
0 20 40 60 80 100
Prevalence (%)

C

Study ID	Cases	Total	Prevalence	95% C.I.	Weight
**Central obesity**					
Zain 2019	1	60	1.7	[0.0; 4.9]	6.7%
Sheikh 2021	15	202	7.4	[3.8; 11.0]	6.7%
Ahmed 2020	1188	15590	7.6	[7.2; 8.0]	6.8%
Malik 2020	115	509	22.6	[19.0; 26.2]	6.7%
Hamid 2010	47	150	31.3	[23.9; 38.8]	6.6%
Shahzad 2017	48	137	35.0	[27.0; 43.0]	6.6%
Shafique 2013	382	1070	35.7	[32.8; 38.6]	6.7%
Arif 2021	113	288	39.2	[33.6; 44.9]	6.7%
Hussain 2016	1708	4319	39.5	[38.1; 41.0]	6.8%
Jahan 2007	101	250	40.4	[34.3; 46.5]	6.7%
Shaikh 2020	110	255	43.1	[37.1; 49.2]	6.7%
Ahsan 2015	20	40	50.0	[34.5; 65.5]	6.2%
Alvi 2011	533	856	62.3	[59.0; 65.5]	6.7%
Ali 2012	934	1329	70.3	[67.8; 72.7]	6.7%
Memon 2020	198	276	71.7	[66.4; 77.1]	6.7%
**Random effects model**		**25331**	**37.1**	**[23.7; 50.5]**	**100.0%**

Heterogeneity: $I^2 = 100\%$, $\tau^2 = 0.0693$, $\chi^2_{14} = 6004.09$ ($p = 0$)

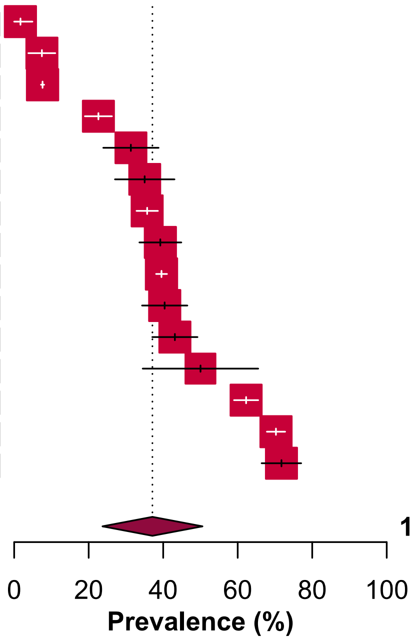

D

Study ID	Cases	Total	Prevalence	95% C.I.	Weight
**High blood pressure**					
Ahmed 2020	835	15590	5.4	[5.0; 5.7]	6.8%
Sheikh 2021	22	202	10.9	[6.6; 15.2]	6.7%
Malik 2020	74	509	14.5	[11.5; 17.6]	6.8%
Hussain 2016	953	4319	22.1	[20.8; 23.3]	6.8%
Hamid 2010	35	150	23.3	[16.6; 30.1]	6.6%
Jahan 2007	62	250	24.8	[19.4; 30.2]	6.7%
Shaikh 2020	64	255	25.1	[19.8; 30.4]	6.7%
Arif 2021	82	288	28.5	[23.3; 33.7]	6.7%
Ahsan 2015	13	40	32.5	[18.0; 47.0]	5.9%
Shafique 2013	381	1070	35.6	[32.7; 38.5]	6.8%
Shahzad 2017	52	137	38.0	[29.8; 46.1]	6.5%
Memon 2020	108	276	39.1	[33.4; 44.9]	6.7%
Riaz 2011	133	337	39.5	[34.2; 44.7]	6.7%
Alvi 2011	420	856	49.1	[45.7; 52.4]	6.8%
Ali 2012	729	1329	54.9	[52.2; 57.5]	6.8%
**Random effects model**		**25608**	**29.5**	**[20.0; 38.9]**	**100.0%**

Heterogeneity: $I^2 = 100\%$, $\tau^2 = 0.0341$, $\chi^2_{14} = 3291.76$ ($p = 0$)

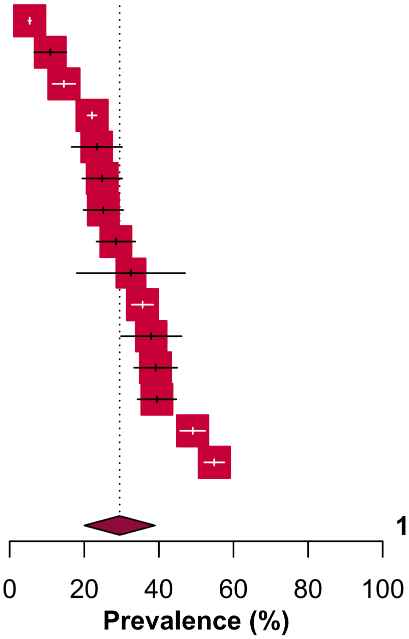

E

Study ID	Cases	Total	Prevalence	95% C.I.	Weight
**Increased glucose**					
Malik 2020	10	509	2.0	[0.8; 3.2]	6.6%
Ahmed 2020	536	15590	3.4	[3.2; 3.7]	6.6%
Sheikh 2021	7	202	3.5	[0.9; 6.0]	6.5%
Hussain 2016	242	4319	5.6	[4.9; 6.3]	6.6%
Memon 2020	29	276	10.5	[6.9; 14.1]	6.4%
Alvi 2011	95	856	11.1	[9.0; 13.2]	6.6%
Shafique 2013	157	1070	14.7	[12.6; 16.8]	6.6%
Ahsan 2015	6	40	15.0	[3.9; 26.1]	5.1%
Arif 2021	50	288	17.4	[13.0; 21.7]	6.3%
Hamid 2010	27	150	18.0	[11.9; 24.1]	6.1%
Zahid 2008	304	1658	18.3	[16.5; 20.2]	6.6%
Zain 2019	13	60	21.7	[11.2; 32.1]	5.3%
Shaikh 2020	63	255	24.7	[19.4; 30.0]	6.2%
Shahzad 2017	58	137	42.3	[34.1; 50.6]	5.7%
Ali 2012	834	1329	62.8	[60.2; 65.4]	6.5%
Jahan 2007	160	250	64.0	[58.0; 70.0]	6.1%
**Random effects model**		**26989**	**20.6**	**[15.3; 25.9]**	**100.0%**

Heterogeneity: I^2 = 99%, τ^2 = 0.0111, χ^2_{15} = 2927.85 (p = 0)

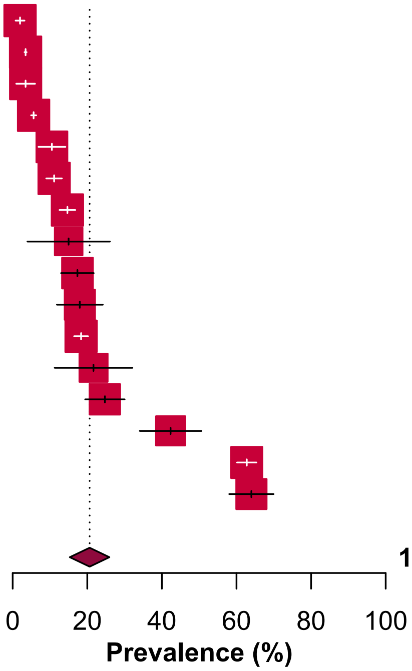

F

Study ID	Cases	Total	Prevalence	95% C.I.	Weight
**High TG**					
Ahmed 2020	647	15590	4.2	[3.8; 4.5]	6.4%
Malik 2020	42	509	8.3	[5.9; 10.6]	6.3%
Sheikh 2021	18	202	8.9	[5.0; 12.8]	6.3%
Shafique 2013	263	1070	24.6	[22.0; 27.2]	6.3%
Shaikh 2020	63	255	24.7	[19.4; 30.0]	6.3%
Alvi 2011	217	856	25.4	[22.4; 28.3]	6.3%
Zahid 2008	487	1658	29.4	[27.2; 31.6]	6.3%
Ahsan 2015	13	40	32.5	[18.0; 47.0]	5.8%
Hamid 2010	52	150	34.7	[27.1; 42.3]	6.2%
Memon 2020	97	276	35.1	[29.5; 40.8]	6.3%
Arif 2021	103	288	35.8	[30.2; 41.3]	6.3%
Ali 2012	532	1329	40.0	[37.4; 42.7]	6.3%
Hussain 2016	2044	4319	47.3	[45.8; 48.8]	6.3%
Jahan 2007	150	250	60.0	[53.9; 66.1]	6.2%
Shahzad 2017	104	137	75.9	[68.8; 83.1]	6.2%
Zain 2019	53	60	88.3	[80.2; 96.5]	6.2%
**Random effects model**		**26989**	**35.8**	**[24.3; 47.3]**	**100.0%**

Heterogeneity: I^2 = 100%, τ^2 = 0.0541, χ^2_{15} = 5773.24 (p = 0)

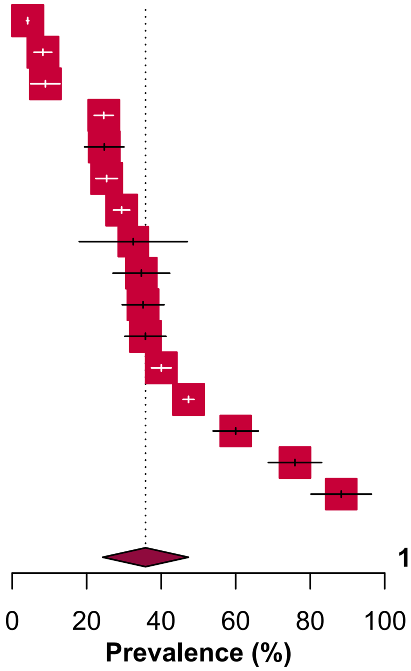

G

Study ID	Cases	Total	Prevalence	95% C.I.	Weight
**Low HDL**					
Sheikh 2021	11	202	5.4	[2.3; 8.6]	5.9%
Ahmed 2020	981	15590	6.3	[5.9; 6.7]	5.9%
Hamid 2010	36	150	24.0	[17.2; 30.8]	5.9%
Shaikh 2020	85	255	33.3	[27.5; 39.1]	5.9%
Shafique 2013	395	1070	36.9	[34.0; 39.8]	5.9%
Arif 2021	108	288	37.5	[31.9; 43.1]	5.9%
Jahan 2007	101	250	40.4	[34.3; 46.5]	5.9%
Zain 2019	32	60	53.3	[40.7; 66.0]	5.7%
Ali 2012	715	1329	53.8	[51.1; 56.5]	5.9%
Ahsan 2015	22	40	55.0	[39.6; 70.4]	5.7%
Hussain 2016	2538	4319	58.8	[57.3; 60.2]	5.9%
Alvi 2011	509	856	59.5	[56.2; 62.8]	5.9%
Malik 2020	311	509	61.1	[56.9; 65.3]	5.9%
Memon 2020	175	276	63.4	[57.7; 69.1]	5.9%
Riaz 2011	233	337	69.1	[64.2; 74.1]	5.9%
Zahid 2008	1320	1658	79.6	[77.7; 81.6]	5.9%
Shahzad 2017	113	137	82.5	[76.1; 88.8]	5.9%
**Random effects model**		**27326**	**48.2**	**[30.8; 65.6]**	**100.0%**

Heterogeneity: I^2 = 100%, τ^2 = 0.1322, χ^2_{16} = 13310.73 (p = 0)

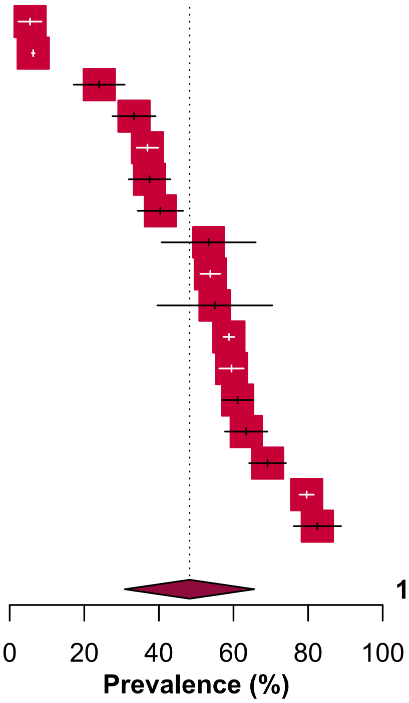

Figure S1. Subgroup analyses based on the diagnostic criteria (A-B) and types of metabolic syndrome (C-G).
