# Supplementary material for: Prevalence of Metabolic Syndrome among Apparently Healthy Adult Population in Pakistan: A Systematic Review and Meta-Analysis
Source: Healthcare (Basel). 2023 Feb 10;11(4):531. doi: 10.3390/healthcare11040531 (PMC9957355; doi:10.3390/healthcare11040531)
Supplement: Supplementary file 1 [file healthcare-11-00531-s001.zip › Figure S2_Sensitivity analyses.pdf]

A

Study ID	Cases	Total	Prevalence	95% C.I.	Weight
**Excluding outlier studies**					
Malik 2020	31	509	6.1	[4.0; 8.2]	5.8%
Sheikh 2021	15	202	7.4	[3.8; 11.0]	5.7%
Ahmed 2020	1188	15590	7.6	[7.2; 8.0]	5.8%
Shaikh 2020	32	255	12.5	[8.5; 16.6]	5.7%
Hydrie 2009	126	867	14.5	[12.2; 16.9]	5.8%
Shafique 2012	301	2032	14.8	[13.3; 16.4]	5.8%
Alam 2011	29	194	14.9	[9.9; 20.0]	5.6%
Hamid 2010	25	150	16.7	[10.7; 22.6]	5.5%
Shafique 2013	192	1070	17.9	[15.6; 20.2]	5.8%
Zain 2019	12	60	20.0	[9.9; 30.1]	5.0%
Ahsan 2015	9	40	22.5	[9.6; 35.4]	4.5%
Riaz 2011	108	337	32.0	[27.1; 37.0]	5.6%
Alvi 2011	298	856	34.8	[31.6; 38.0]	5.7%
Jahan 2007	88	250	35.2	[29.3; 41.1]	5.5%
Arif 2021	113	288	39.2	[33.6; 44.9]	5.5%
Zahid 2008	679	1658	41.0	[38.6; 43.3]	5.8%
Memon 2020	128	276	46.4	[40.5; 52.3]	5.5%
Shahzad 2017	82	137	59.9	[51.6; 68.1]	5.2%
**Random effects model**		**24771**	**24.4**	**[18.6; 30.2]**	**100.0%**

Heterogeneity: I^2 = 99%, τ^2 = 0.0149, χ^2_{17} = 1678.38 (p = 0)

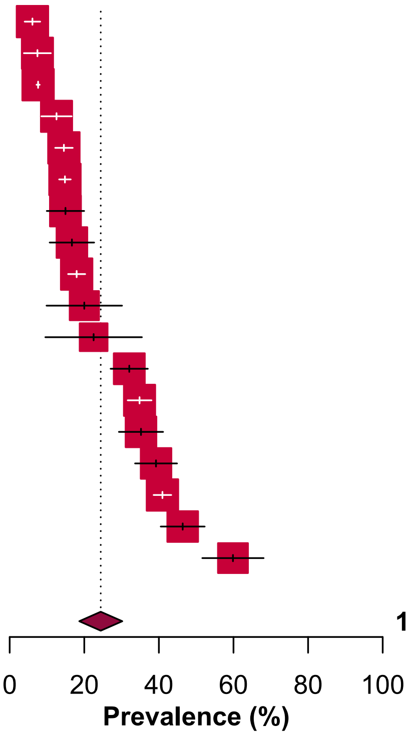

B

Study ID	Cases	Total	Prevalence	95% C.I.	Weight
**Excluding small studies (n<100)**					
Malik 2020	31	509	6.1	[4.0; 8.2]	5.6%
Sheikh 2021	15	202	7.4	[3.8; 11.0]	5.6%
Ahmed 2020	1188	15590	7.6	[7.2; 8.0]	5.6%
Shaikh 2020	32	255	12.5	[8.5; 16.6]	5.6%
Hydrie 2009	126	867	14.5	[12.2; 16.9]	5.6%
Shafique 2012	301	2032	14.8	[13.3; 16.4]	5.6%
Alam 2011	29	194	14.9	[9.9; 20.0]	5.5%
Hamid 2010	25	150	16.7	[10.7; 22.6]	5.5%
Shafique 2013	192	1070	17.9	[15.6; 20.2]	5.6%
Riaz 2011	108	337	32.0	[27.1; 37.0]	5.5%
Alvi 2011	298	856	34.8	[31.6; 38.0]	5.6%
Jahan 2007	88	250	35.2	[29.3; 41.1]	5.5%
Arif 2021	113	288	39.2	[33.6; 44.9]	5.5%
Zahid 2008	679	1658	41.0	[38.6; 43.3]	5.6%
Memon 2020	128	276	46.4	[40.5; 52.3]	5.5%
Shahzad 2017	82	137	59.9	[51.6; 68.1]	5.4%
Ali 2012	847	1329	63.7	[61.1; 66.3]	5.6%
Hussain 2016	2935	4319	68.0	[66.6; 69.3]	5.6%
**Random effects model**		**30319**	**29.6**	**[18.0; 41.1]**	**100.0%**

Heterogeneity: I^2 = 100%, τ^2 = 0.0617, χ^2_{17} = 9223.72 (p = 0)

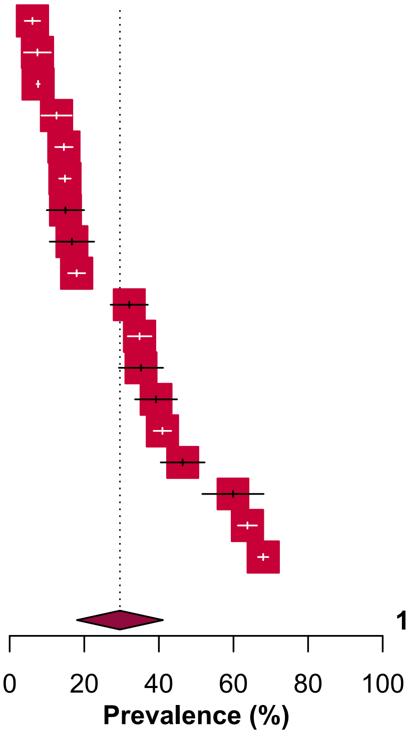

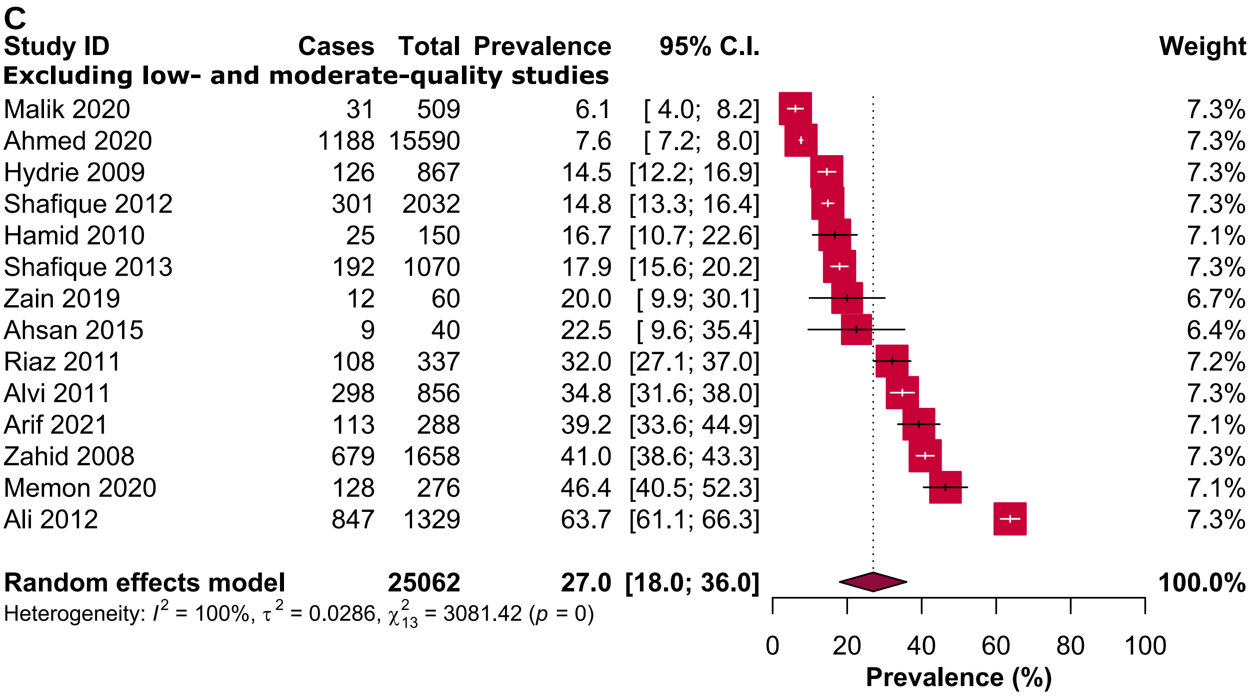

Figure S2. Sensitivity analyses.
