# Supplementary material for: Prevalence of Metabolic Syndrome among Apparently Healthy Adult Population in Pakistan: A Systematic Review and Meta-Analysis
Source: Healthcare (Basel). 2023 Feb 10;11(4):531. doi: 10.3390/healthcare11040531 (PMC9957355; doi:10.3390/healthcare11040531)
Supplement: Supplementary file 1 [file healthcare-11-00531-s001.zip › Table S1.pdf]

**Table S1: Search strategy used for PubMed**

| Search Terms (No. of Hits) |
|---|
| **PubMed (n=103)** |
| ("metabolic syndrome"[Title] OR "metabolic syndromes"[Title] OR "insulin resistance syndrome"[Title]) AND (Pakistan[Title/Abstract] OR Sindh[Title/Abstract] OR Punjab[Title/Abstract] OR Balochistan[Title/Abstract] OR "Khyber Pakhtunkhwa"[Title/Abstract] OR KPK[Title/Abstract] OR NWFP[Title/Abstract] OR Gilgit[Title/Abstract] OR "Gilgit Baltistan"[Title/Abstract] OR "GilgitBaltistan"[Title/Abstract] OR "Azad Kashmir"[Title/Abstract] OR Karachi[Title/Abstract] OR Hyderabad[Title/Abstract] OR Lahore[Title/Abstract] OR Islamabad[Title/Abstract] OR Rawalpindi[Title/Abstract] OR Sialkot[Title/Abstract] OR Sargodha[Title/Abstract] OR Multan[Title/Abstract] OR Gujrat[Title/Abstract] OR Quetta[Title/Abstract] OR Faisalabad[Title/Abstract] OR Gujranwala[Title/Abstract] OR Bahawalpur[Title/Abstract] OR Bahawalnagar[Title/Abstract] OR Chakwal[Title/Abstract] OR Kasur[Title/Abstract] OR Peshawar[Title/Abstract] OR "Dera Ismail Khan"[Title/Abstract] OR "DI Khan"[Title/Abstract] OR Swat[Title/Abstract] OR FATA[Title/Abstract] OR Abbottabad[Title/Abstract] OR Banu[Title/Abstract] OR Buner[Title/Abstract] OR "Dir lower"[Title/Abstract] OR Hangu[Title/Abstract] OR Haripur[Title/Abstract] OR Karak[Title/Abstract] OR Kohat[Title/Abstract] OR "Lakki Marwat"[Title/Abstract] OR Malakand[Title/Abstract] OR Mansehra[Title/Abstract] OR Mardan[Title/Abstract] OR Nowshera[Title/Abstract] OR Swabi[Title/Abstract] OR Shikarpur[Title/Abstract] OR Rohri[Title/Abstract] OR Larkana[Title/Abstract] OR Thatta[Title/Abstract]) |
| **SCOPUS (n=155)** |
| TITLE-ABS("metabolic syndrome" OR "metabolic syndromes" OR "insulin resistance syndrome") AND TITLE-ABS(Pakistan OR Sindh OR Punjab OR Balochistan OR "Khyber Pakhtunkhwa" OR KPK OR NWFP OR Gilgit OR "Gilgit Baltistan" OR "Gilgit-Baltistan" OR "Azad Kashmir" OR Karachi OR Hyderabad OR Lahore OR Islamabad OR Rawalpindi OR Sialkot OR Sargodha OR Multan OR Gujrat OR Quetta OR Faisalabad OR Gujranwala OR Bahawalpur OR Bahawalnagar OR Chakwal OR Kasur OR Peshawar OR "Dera Ismail Khan" OR "DI Khan" OR Swat OR FATA OR Abbottabad OR Banu OR Buner OR "Dir lower" OR Hangu OR Haripur OR Karak OR Kohat OR "Lakki Marwat" OR Malakand OR Mansehra OR Mardan OR Nowshera OR Swabi OR Shikarpur OR Rohri OR Larkana OR Thatta) |
| **ScienceDirect (n=14)** |
| ("metabolic syndrome" OR "metabolic syndromes" OR "insulin resistance syndrome" AND Pakistan) |
| **Google Scholar (n=34)** |
| "metabolic syndrome" OR "metabolic syndromes" OR "insulin resistance syndrome" Pakistan |
| **Web of Science (n=134)** |
| (TI, AB=("metabolic syndrome" OR "metabolic syndromes" OR "insulin resistance syndrome")) AND TI, AB=(Pakistan OR Sindh OR Punjab OR Balochistan OR "Khyber Pakhtunkhwa" OR KPK OR NWFP OR Gilgit OR "Gilgit Baltistan" OR "Gilgit-Baltistan" OR "Azad Kashmir" OR Karachi OR Hyderabad OR Lahore OR Islamabad OR Rawalpindi OR Sialkot OR Sargodha OR Multan OR Gujrat OR Quetta OR Faisalabad OR Gujranwala OR Bahawalpur OR Bahawalnagar OR Chakwal OR Kasur OR Peshawar OR "Dera Ismail Khan" OR "DI Khan" OR Swat OR FATA OR Abbottabad OR Banu OR Buner OR "Dir lower" OR Hangu OR Haripur OR Karak OR Kohat OR "Lakki Marwat" OR Malakand OR Mansehra OR Mardan OR Nowshera OR Swabi OR Shikarpur OR Rohri OR Larkana OR Thatta) |
