# Supplementary material for: Prevalence of Metabolic Syndrome among Apparently Healthy Adult Population in Pakistan: A Systematic Review and Meta-Analysis
Source: Healthcare (Basel). 2023 Feb 10;11(4):531. doi: 10.3390/healthcare11040531 (PMC9957355; doi:10.3390/healthcare11040531)
Supplement: Supplementary file 1 [file healthcare-11-00531-s001.zip › Table S2.pdf]

Table S2: Criteria for diagnosis of metabolic syndrome

	**Metabolic Syndrome**	**Waist Circumference (WC)**	**Blood Pressure (BP)**	**Fasting Plasma Glucose**	**Triglyceride (TG)**	**HDL-C**
**NCEP-ATP III (2001)**	*Presence of at least 3 RF*	For Males ≥102cm, For Females ≥88cm	SBP ≥130 mm/Hg and/or DBP ≥85 mm/Hg	≥6.1 mmol/L	≥1.7 mmol/L	For Males <1.03 mmol/L, For Females < 1.29 mmol/L
**IDF (2005)**	*WC with 2 or RF*	For Males ≥90cm, For Females ≥80cm	SBP ≥130 mm/Hg and/or DBP ≥85 mm/Hg or on treatment for hypertension	≥5.6 mmol/L or previously diagnosed T2DM	≥1.7 mmol/L or on treatment for TG	For Males <1.03 mmol/L, For Females < 1.29 mmol/L or on treatment for HDL-C
**Modified NCEP-ATP III (2005)**	*Presence of at least 3 RF*	For Males ≥90cm, For Females ≥80cm	SBP ≥130 mm/Hg and/or DBP ≥85 mm/Hg or on treatment for hypertension	≥5.6 mmol/L or previously elevated glucose	≥1.7 mmol/L or on treatment for TG	For Males <1.03 mmol/L, For Females < 1.29 mmol/L or on treatment for HDL-C
**JIS (2009)**	*Presence of at least 3 RF*	For Males ≥90cm, For Females ≥80cm	SBP ≥130 mm/Hg and/or DBP ≥85 mm/Hg or on treatment for hypertension	≥5.6 mmol/L or previously elevated glucose	≥1.7 mmol/L or on treatment for TG	For Males <1.03 mmol/L, For Females < 1.3 mmol/L or on treatment for HDL-C
