# Supplementary material for: Prevalence of Metabolic Syndrome among Apparently Healthy Adult Population in Pakistan: A Systematic Review and Meta-Analysis
Source: Healthcare (Basel). 2023 Feb 10;11(4):531. doi: 10.3390/healthcare11040531 (PMC9957355; doi:10.3390/healthcare11040531)
Supplement: Supplementary file 1 [file healthcare-11-00531-s001.zip › Table S3_Quality assessment_Case control.pdf]

Table S3. Quality assessment of the included case-control studies

No.	Study ID	Questions assessing the case-control studies										Yes (%)
		1	2	3	4	5	6	7	8	9	10	
1	Hamid 2010 (21)	Y	Y	Y	U	Y	Y	N	Y	Y	N	70.0
2	Sheikh 2021 (30)	Y	Y	Y	Y	Y	N	N	N	Y	N	60.0
3	Zain 2019 (32)	Y	Y	Y	Y	Y	N	N	Y	Y	N	70.0

1. Were the groups comparable other than the presence of disease in case or the absence of disease in controls? 2. Were cases and controls matched appropriately? 3. Were the same criteria used for identification of cases and controls? 4. Was exposure measured in a standard, valid and reliable way? 5. Was exposure measured in the same way for cases and controls? 6. Were confounding factors identified? 7. Were strategies to deal with confounding factors stated? 8. Were outcomes assessed in a standard, valid and reliable way for cases and controls? 9. Was the exposure period of interest long enough to be meaningful? 10. Was appropriate statistical analysis used? Y=Yes; N=No; U=Unclear.
