# Supplementary material for: Prevalence of Metabolic Syndrome among Apparently Healthy Adult Population in Pakistan: A Systematic Review and Meta-Analysis
Source: Healthcare (Basel). 2023 Feb 10;11(4):531. doi: 10.3390/healthcare11040531 (PMC9957355; doi:10.3390/healthcare11040531)
Supplement: Supplementary file 1 [file healthcare-11-00531-s001.zip › Table S4_Quality assessment_Cross sectional.pdf]

Table S4. Quality assessment of the included cross-sectional studies

No.	Study ID	Questions assessing the cross-sectional studies								Yes (%)
		1	2	3	4	5	6	7	8	
1	Ahmed 2020 (10)	Y	Y	Y	Y	Y	Y	Y	Y	100
2	Ahsan 2015 (16)	N	Y	Y	Y	Y	Y	Y	Y	87.5
3	Alam 2011 (17)	Y	N	Y	Y	N	N	Y	N	50.0
4	Ali 2012 (18)	Y	Y	Y	Y	Y	Y	Y	Y	100
5	Alvi 2011 (19)	Y	Y	Y	Y	Y	Y	Y	Y	100
6	Arif 2021 (20)	Y	N	Y	Y	Y	Y	Y	N	75.0
7	Hussain 2016 (11)	N	N	Y	Y	Y	Y	Y	U	62.5
8	Hydrie 2009 (9)	Y	Y	Y	Y	Y	Y	N	N	75.0
9	Jahan 2007 (22)	Y	Y	Y	Y	N	N	Y	N	62.5
10	Malik 2020 (23)	Y	Y	Y	Y	Y	Y	Y	Y	100
11	Memon 2020 (24)	Y	N	Y	Y	Y	Y	Y	N	75.0
12	Riaz 2011 (25)	Y	N	Y	Y	Y	Y	Y	Y	87.5
13	Shafique 2012 (26)	Y	Y	Y	Y	Y	Y	Y	Y	100
14	Shafique 2013 (27)	Y	Y	Y	Y	Y	Y	Y	Y	100
15	Shaikh 2020 (28)	N	N	Y	N	N	N	Y	N	25.0
16	Shahzad 2017 (29)	N	N	N	N	N	N	Y	N	12.5
17	Zahid 2008 (31)	N	N	Y	Y	Y	Y	Y	Y	75.0

1. Were the criteria for inclusion in the sample clearly defined? 2. Were the study subjects and the setting described in detail? 3. Was the exposure measured in a valid and reliable way? 4. Were objective, standard criteria used for measurement of the condition? 5. Were confounding factors identified? 6. Were strategies to deal with confounding factors stated? 7. Were the outcomes measured in a valid and reliable way? 8. Was appropriate statistical analysis used? Y=Yes; N=No; U=Unclear.
